# Materials for Wind Turbine Blades: An Overview

**DOI:** 10.3390/ma10111285

**Published:** 2017-11-09

**Authors:** Leon Mishnaevsky, Kim Branner, Helga Nørgaard Petersen, Justine Beauson, Malcolm McGugan, Bent F. Sørensen

**Affiliations:** Department of Wind Energy, Technical University of Denmark, 4000 Roskilde, Denmark; kibr@dtu.dk (K.B.); hnpe@dtu.dk (H.N.P.); jube@dtu.dk (J.B.); mamc@dtu.dk (M.M.);bsqr@dtu.dk (B.F.S.)

**Keywords:** wind energy, composite materials, properties, reliability, modeling, manufacturing, wind turbine, blades

## Abstract

A short overview of composite materials for wind turbine applications is presented here. Requirements toward the wind turbine materials, loads, as well as available materials are reviewed. Apart from the traditional composites for wind turbine blades (glass fibers/epoxy matrix composites), natural composites, hybrid and nanoengineered composites are discussed. Manufacturing technologies for wind turbine composites, as well their testing and modelling approaches are reviewed.

## 1. Introduction

The reduction of fossil fuel dependency requires the expansion of the renewable energy sector. The European Union seeks to cover 20% of its energy needs from renewables by 2020. In order to achieve this goal [1,2], the wind energy capacity should be expanded by two orders of magnitude. The EU offshore wind energy capacity is expected to grow by 21% annually [3,4,5].

**Figure 1:**
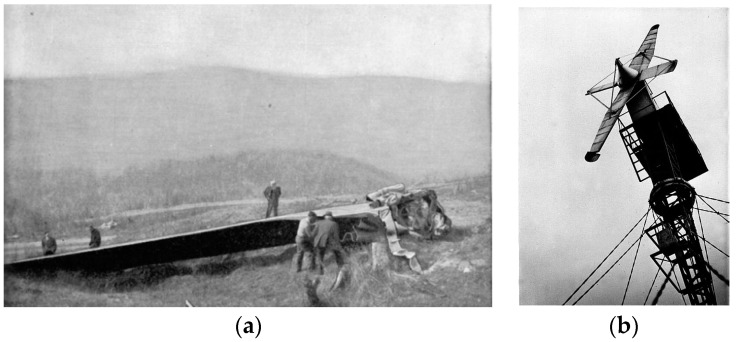
Early history of wind turbines: (**a**) Failed blade of Smith wind turbine of 1941 (Reprinted from [10]; and (**b**) Gedser wind turbine (from [11]).

The history of wind turbines for electric power generation started in 1988 Cleveland Ohio, USA, 1888 by Charles F. Brush [6] and in Askov, Denmark in 1889 by pioneer Poul La Cour [7]. In 1941, electricity production from wind was made using turbines with steel blades built by the company S. Morgan-Smith at Grandpa’s Knob in Vermont in USA. One of the blades failed after only a few hundred hours of intermittent operation (see Figure 1a). Thus, the importance of the proper choice of materials and inherent limitations of metals as a wind blade material was demonstrated early in the history of wind energy development. The next, quite successful example of the use of the wind turbine for energy generation is the so-called Gedser wind turbine, designed by Johannes Juul, with three composite blades built from steel spars, with aluminum shells supported by wooden ribs, installed at Gedser coast in Denmark in 1956–1957. After the 1970s, wind turbines were mainly produced with composite blades [8,9].

The Gedser turbine (three blades, 24 m rotor, 200 kW, Figure 1b) was the first success story of wind energy, running for 11 years without maintenance. In this way, the linkage between the success of wind energy generation technology and the application of composite materials became an issue from the beginning: the first turbine, built with steel blades, failed, while the second one, with composite blades, worked for many years. 

## 2. Composite Structures of Wind Turbines: Loads and Requirements 

### 2.1. Overview of Blade Design

Composite materials are used typically in blades and nacelles of wind turbines. Generator, tower, etc. are manufactured from metals. Blades are the most important composite based part of a wind turbine, and the highest cost component of turbines. 

**Figure 2:**
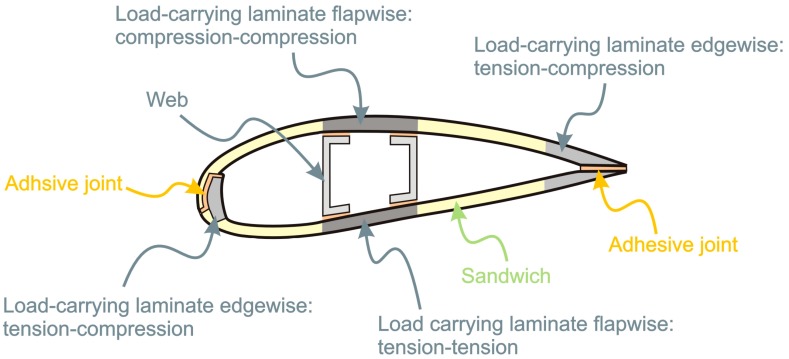
Schema of the section of the blade.

A wind turbine blades consists of two faces (on the suction side and the pressure side), joined together and stiffened either by one or several integral (shear) webs linking the upper and lower parts of the blade shell or by a box beam (box spar with shell fairings) (see Schema on Figure 2) [12]. The flapwise load is caused by the wind pressure, and the edgewise load is caused by gravitational forces and torque load. The flapwise bending is resisted by the spar, internal webs or spar inside the blade, while the edges of the profile carry the edgewise bending. From the point of loads on materials, one of the main laminates in the main spar is subjected to cyclic tension-tension loads (pressure side) while the other (suction side) is subjected to cyclic compression-compression loads. The laminates at the leading and trailing edges that carry the bending moments associated with the gravitation loads are subjected to tension-compression loads. The aeroshells, which are made of sandwich structures, are primarily designed against elastic buckling. The different cyclic loading histories that exist at the various locations at the blades suggest that it could be advantageous to use different materials for different parts of the blade. 

A major trend in wind turbine development is the increase in size and offshore placements. Increasing size is motivating by the desire to reduce of the leveraged cost of energy. With increasing size, the weight of the rotor blades increases, so that gravitational loads become design drivers. Also longer blades deflect more, so that structural stiffness (to ensure tip clearance, i.e., to avoid the blade to hit the tower) is of increasing importance. Thus, from a materials perspective, the stiffness-to-weight is of major importance. In addition, with the turbine designed to be in operation for 20–25 years, the high-cycle fatigue (exceeding 100 million load cycles) behavior of composites and material interfaces (bondlines, sandwich/composite interfaces) is of major importance. 

### 2.2. Overview of Manufacturing of Wind Turbine Blades

During the first decades of the wind energy development, wind turbine blades were often produced using the wet hand lay-up technology, in open molds. The glass-fiber reinforcement was impregnated using paint brushes and rollers. The shells were adhesively bonded together/to the spars. This technology was used mainly to produce small and medium size blades (up to 35 and 55 m, respectively). For larger blades, the same technology was used, but the web were inserted and adhesively bonded between two sides, and the plies with more fiber content were used. The disadvantages of the open mold technology are high labor costs, relatively low quality of products and environmental problems. In 1970s, several companies and institutes explored the applicability of filament winding technology, seeking to improve the quality of turbine and to reduce labor costs [13].

The introduction of vacuum infusion and prepreg technologies allowed improving the quality of manufacturing [14]. The prepreg technology, adapted from the aircraft industry, is based on utilizing “pre-impregnated” composite fibers, which already contain an amount of the matrix material bonding them together. Prepreg (widely used, for instance, by the Danish wind turbine producer Vestas) allows the industrial impregnation of fibers, and then forming the impregnated fibers to complex shapes. 

**Figure 3:**
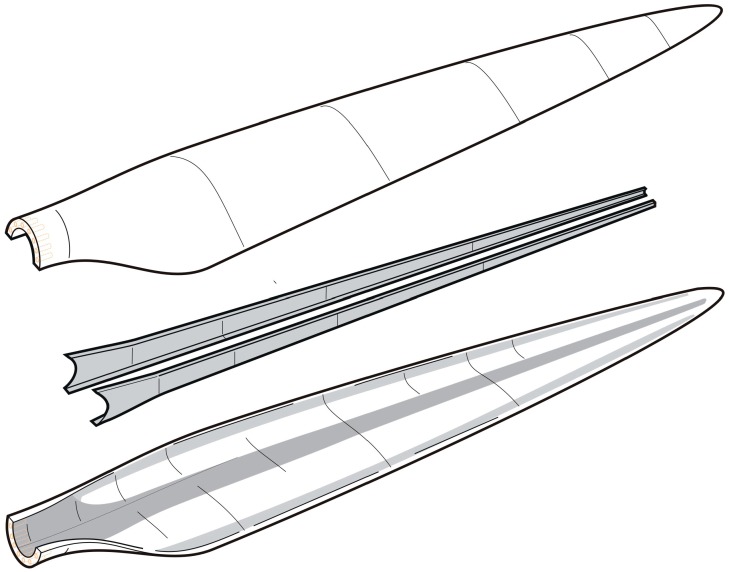
Schematics of the manufacturing of a wind turbine rotor blade by assemblage and bonding of two aeroshells and two shear webs (gray color indicates the primary load-carrying composites).

The most widely used technology to produce the wind blades, especially longer blades, is the resin infusion technology. In the resin infusion technology, fibers are placed in closed and sealed mold, and resin is injected into the mold cavity under pressure. After the resin fills all the volume between fibers, the component is cured with heat. The resin infusion technologies can be divided into two groups: Resin Transfer Molding (RTM) (resin injection under pressure higher than atmospheric one) and Vacuum Assisted Resin Transfer Molding (VARTM) (or Vacuum Infusion Process) (when resin is injected under vacuum or pressure lower than atmospheric, typically, under a vacuum bag) [15]. A variation of VARTM called SCRIMP™ (i.e., Seemann Composite Resin Infusion Process) was developed in late 1980s and is quite efficient for producing large and thick parts. Currently, vacuum assisted resin transfer molding (VARTM) is the most common manufacturing method for manufacturing of wind turbine rotor blades. With his method, layers of fabrics of dry fibers, with nearly all unidirectional fibers, aligned in the direction along the length of the blade, are position on mold parts along with polymer foams or balsa wood for sandwich structures (for the aeroshells). In order to form a laminate that is thick by the root and gradually becomes thinner towards the tip, most plies run from the root only partly toward the tip; the termination of a ply is called ply-drop. The fabrics and subsequently covered by a vacuum bag and made air-tight. After the application of vacuum, low-viscosity resin flows in and wets the fibers. After infusion, the resin cures at room temperature. In most cases, wind turbine rotor blades are made in large parts, e.g., as two aeroshells with a load-carrying box (spar) or internal webs that are then bonded together. Sometimes, the composite structure is post cured at elevated temperature. In principle, this manufacturing method is well suited for upscaling, since the number of resin inlets and vacuum suction points can be increased. A challenge with upscaling is however, than quite many layer of dry fabrics must be kept in place and should not slip relative to each other. The composite is quite thick by the root section, typically exceeding 50–60 mm in the consolidated state. In practice, it can be a challenge to avoid the formation of wrinkles at double-curved areas and areas with un-wetted fibers and air bubbles can be entrapped in the bondlines. After manufacturing, the blades are subjected to quality control and manufacturing defects are repaired. Since a large blade represents a large value in materials, increasing sizes means that it becomes less and less attractive to discard blades with manufacturing defects. Thus, with increasing size the requirements towards materials go towards easier processing and materials should preferably be more damage tolerant so that larger manufacturing defects can be tolerated. Figure 3 shows the schematics of the manufacturing of a wind turbine rotor blade by assemblage and bonding of two aeroshells and two shear webs.

The infusion process is usually cheaper that the prepreg process. However, the prepreg composites have more stable, better and less variable mechanical properties than the composites produced by resin infusion. This technology is relatively environmental friendly, and makes it possible to achieve higher volume content of fibers, and to control the materials properties. Further, the prepreg technology allows higher level of automation and better choice of resins. 

Lately, the automated tape lay-up, automated fiber placement, two-pieces or segment wind blades, enhanced finishing technologies are expected to come into use to improve quality and reduce costs of the composite blade manufacturing [14]. A big challenge, in comparison with e.g., automatization of composite structures for aerospace, is the much larger thicknesses and the much larger amount of materials to be places in the molds for wind turbine rotor blades. For some parts of the blades, 3D woven composites represent a promising alternative to producing fiber reinforced laminates. Mohamed and Wetzel [16] suggested producing spar caps from 3D woven carbon/glass hybrid composites. It was demonstrated that this technology allows producing spar caps with higher stiffness and lower weight, than the commonly used technologies. 

### 2.3. Overview of Blade Damage

Precise information about the range and extent of damages found and repaired in operating wind farms is not generally available, however detailed studies of the composite material and adhesive interface damage found in wind turbine blades subjected to structural testing have existed for some time [17,18]. The static loads and cyclic loading applied to the blade structure during full scale testing can result in the damage in the form of failure of various adhesive layers, laminate delamination, debonding at skin/core interfaces and splitting along fibers or in-plane compressive failure as well as gelcoat/skin debonding and cracks in the gelcoat. Obviously, damages in the primary load-carrying laminates (main spar and laminates at the leading and trailing edges) are of major concern. Fortunately composite materials are damage tolerant materials. Still, a major issue is that many of these damage modes are not easily detectable, since the damage do not originate from the external surfaces and may not be visible. For instance, in thick composite parts wrinkles may lead to the formation of compression failure and delamination. Cracks and delamination can also start from processing details such as ply-drops that locally causes a stress concentration. Cracks at e.g., trailing edge bondlines can be seen visually, but it is more difficult to assess how far they extend into the composite structure. 

In addition to the various structural loading effects, wind turbine blades can also be subjected to lightning strikes, physical impacts and damaging surface erosion conditions whilst in operation. In certain rare but dramatic cases, a particular event can cause the total failure of a blade almost immediately; a powerful lightning strike or an extreme wind loading that leads to a rotating blade hitting the tower for example. Operators of wind farms take measures to minimize exposure of their structural assets to the full effect of storm conditions when these are forecast. But more commonly, over the course of a normal 25-year service life, it is expected that the composite material in a wind turbine blade will accumulate some signs of damage.

Blades are the most vulnerable parts of a wind turbine with respect to lightning. As every turbine can expect to experience a significant number of strikes during service life [19], all blades have a lightning protection system to reduce the effect of such strikes when they occur. Despite this it is common to observe scorching damage and cracking around the lightning attraction point of a blade as well as spar rupture, separation and surface tearing in more extreme cases [20].

A significant damage form observed in operating turbine blades is caused by (abrasive) airborne particulates impacting and eroding the leading edge, especially towards the tip where velocities are higher. Once established this rough surface will degrade the aerodynamic performance of the blade and reduce power production; if left unrepaired structural damage to the laminate material will soon develop requiring a longer and more complex repair effort [21,22].

Icing is the accumulation of ice on the surface of the blades under particular low temperature weather conditions [23]. In extreme cases it will stop the operation of the turbine, but before that will disrupt the aerodynamics of the blade and reduce the energy generation as well as unbalancing the load distribution in the system and thus reducing structural fatigue lifetime [24].

## 3. Composites for Wind Turbine Blades

### 3.1. Fibers

***Glass and carbon fibers.*** The stiffness of composites is determined by the stiffness of fibers and their volume content. Typically, E-glass fibers (i.e., borosilicate glass called “electric glass” or “E-glass” for its high electric resistance) are used as main reinforcement in the composites. With increasing the volume content of fibers in UD composites, the stiffness, tensile and compression strength increase proportionally, yet, at high volume content of fibers (after 65%), there might be dry areas without resin between fibers and the fatigue strength of the composite reduces [25]. Typically, the glass/epoxy composites for wind blades contain up to 75 weight % glass.

Many investigations toward the development of fibers, which are stronger than the usual E-glass fibers, have been carried out. The high strength fibers (which are still used seldom in practice, but represent a promising source of the composite materials improvement) include glass fibers with modified compositions (S-glass, R-glass, etc.), carbon fibers, basalt and aramid fibers. S-glass (i.e., high strength glass, S means “Strength” here) developed in the 1960s, shows 40% higher tensile and flexural strengths, and 10–20% higher compressive strength and flexural modulus, as compared to E-glass. The S-glass is much more expensive than E-glass. S2 glass was developed in the 1968 as a commercial version of S-glass. S glass and S2 glass fibers have the same composition (magnesium alumino-silicate). The main differences are in sizing (fiber coating) and certification procedure. The price of S2-glass is around 10 times of that of E-glass. R-Glass fibers, introduced in 1968, are produced with a calcium aluminosilicate glass with less silica and added oxides [26]. Some other special glasses developed by Owens Corning are ECRGLAS, Advantex and most recently WindStrandTM glass fibers. The WindStrandTM glass fibers show15 percent higher stiffness and up to 30 percent higher strength when compared to E-glass [27]. 

Carbon fibers are considered to be a very promising alternative to the glass fibers. They show much higher stiffness and lower density than the glass fibers, thus, allowing the thinner, stiffer and lighter blades. However, they have relatively low damage tolerance, compressive strength and ultimate strain, and are much more expensive than the E glass fibers [28,29]. Carbon fiber reinforced composites are sensitive to the fiber misalignment and waviness: even small misalignments lead to the strong reduction of compressive and fatigue strength. Carbon fiber composites are used by the companies Vestas (Aarhus, Denmark) and Siemens Gamesa (Zamudio, Spain), often in structural spar caps of large blades [28]. 

***Aramid and basalt fibers.*** Further, an interesting alternative is using non-glass, high strength fibers first of all, aramid and basalt fibers. Aramid (aromatic polyamide) fibers demonstrate high mechanical strength, and are tough and damage tolerant, but have low compressive strength, low adhesion to polymer resins, absorb moisture, and degrade due to the ultraviolet radiation [30]. 

Basalt fibers show good mechanical properties, are 30% stronger, 15–20% stiffer and 8–10% lighter than E-glass, and cheaper than the carbon fibers [28]. The application of basalt fibers in small wind turbines have been demonstrated in [31,32], and the results were very encouraging. In the works [31,32], the basalt fibers were used as hybrids with carbon fibers. 

***Hybrid composites.*** Hybrid reinforcements (E-glass/carbon, E-glass/aramid, etc.) represent an interesting alternative to the pure glass or pure carbon reinforcements. Ong and Tsai [33] demonstrated that the full replacement would lead to 80% weight savings, and cost increase by 150%, while a partial (30%) replacement would lead to only 90% cost increase and 50% weight reduction for 8 m turbine. The world currently longest wind turbine rotor blade, the 88.4 m long blade from LM Wind Power is made of carbon/glass hybrid composites [34].

**Figure 4:**
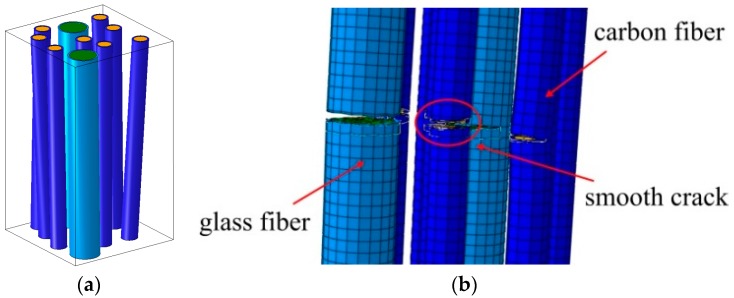
Micromechanical model of hybrid glass/fiber composites (**a**); and the simulated fiber failure (**b**). Reprinted from [41,42] with kind permission from Elsevier.

In a number of works, the strength and damage mechanisms of hybrid composites were studied [35,36,37,38,39,40,41,42,43,44,45,46,47,48,49,50,51,52]. It was reported, among others, that the incorporation of glass fibers in carbon fiber reinforced composites allows the improvement of their impact properties and tensile strain to failure of the carbon fibers. Manders and Bader [40] observed an enhancement of the failure strain of the carbon fiber reinforced phase when “carbon fiber is combined with less-stiff higher-elongation glass fiber in a hybrid composite”. However, in [41], it was shown on the basis of computations that the dependency of the composite strength on the ratio glass/carbon is V-shaped, with a minimum at the content of the order of 60% carbon, i.e., the hybrid strength can be under some conditions be lower than the strength of both pure glass or pure carbon composites. This observation was confirmed experimentally in [43]. Thus, while the hybrid composites seem to be a very promising group of composites for wind energy, additional investigations are required for the optimal composition of the materials. Figure 4 shows a computational micromechanical model of hybrid glass/fiber composites, and the composite degradation process (first, failure of glass fibers and then failure of carbon fibers), as observed in numerical simulations in [41,42].

***Natural fibers.*** In some cases, natural fibers can be used as well. The advantages of natural fibers, as sisal, flax, hemp, jute, are the low costs, availability and environmental friendliness. The disadvantages are the quality variations, high moisture uptake and low thermal stability of the raw fibers [44]. Holmes et al. [45,46] tested a novel bamboo-poplar epoxy laminate for wind turbine blades, and demonstrated that this material has high strength and stiffness, and can be used in wind blades instead of common composites. The high strength and durability of bamboo as well as its quick growth and broad availability make the bamboo to a very promising material for the wind energy applications. 

**Figure 5:**
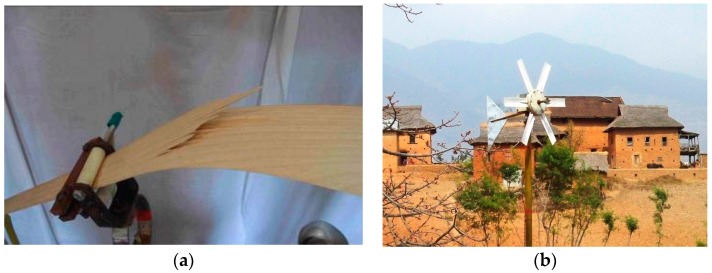
Testing the timber wind turbine blade (**a**) and small wind turbines installed in Nepal (**b**).

An interesting option for developing countries is small turbines, producible on-site, and made from “natural composites”, i.e., from locally available timber [47]. In a series of investigations, a group of Nepali, Danish and Australian scientists studied the applicability of different timbers for wind turbines, and demonstrated that the turbines with wooden blades represent a reliable and low cost option for the developing countries [48,49,50,51]. Figure 5 shows a test of a timber wind turbine blade and small wind turbines installed in a village school in Nepal. 

### 3.2. Matrix

Typically, thermosets (epoxies, polyesters, vinylesthers) or (more seldom) thermoplastics are used as matrices in wind blade composites. 

***Thermosets.*** Thermosets based composites represent around 80% of the market of reinforced polymers [53,54]. The advantages of thermosets are the possibility of room or low temperature cure, and lower viscosity (which eases infusion and thus, allowing high processing speed). Initially, polyester resins were used for composite blades. With the development of large and extra-large wind turbines, epoxy resins replaced polyester and are now used most often as matrices of wind blade composites. Still, recent studies (e.g., by Swiss company DSM Composite Resins) support arguments for the return to unsaturated polyester resins, among them, faster cycle time and improved energy efficiency in the production, stating that the newly developed polyesters meet all the strength and durability requirements for large wind blades. 

Further, the development of matrix materials which cure faster and at lower temperatures is an important research area. 

***Thermoplastics.*** Thermoplastics represent an interesting alternative to the thermoset matrices. The important advantage of thermoplastic composites is their recyclability. Their disadvantages are the necessity of high processing temperatures (causing the increased energy consumption and possibly influencing fiber properties) and, difficulties to manufacture large (over 2 m) and thick (over 5 mm) parts, due to the much higher viscosity. The melt viscosity of thermoplastic matrices is of the order 10^2^–10^3^ Pa s, while that for thermosetting matrix is around 0.1–10 Pa s. Thermoplastics (as differed from thermosets) have melting temperatures lower than their decomposition temperatures, and, thus, can be reshaped upon melting. While the fracture toughness of thermoplastics is higher than that of thermosets, fatigue behavior of thermoplastics is generally not as good as thermosets, both with carbon or glass fibers [53]. Other advantages of thermoplastics include the larger elongation at fracture, possibility of automatic processing, and unlimited shell life of raw materials [55]. 

**Figure 6:**
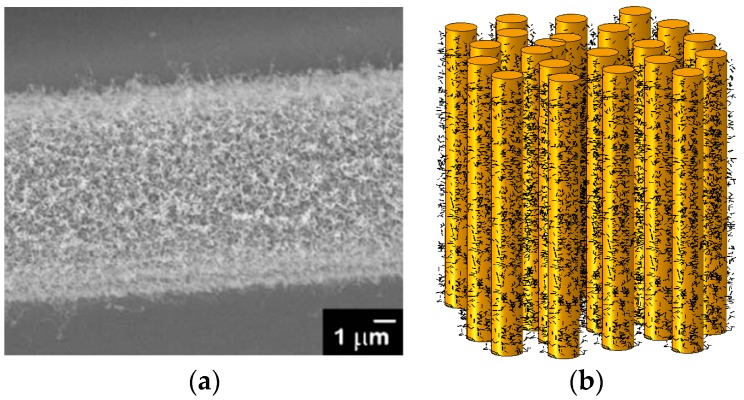
Micrograph of a carbon fiber with CNT reinforcements in fiber-matrix interface (**a**) and computational model of a composite with secondary CNT particles (**b**). (Reprinted from [65,69] with kind permission from Elsevier).

***Nanoengineered polymers and composites.*** In several works, the possibilities of improvement of composites properties by adding nanoreinforcement in matrix were demonstrated. Additions of small amount (at the level of 0.5 weight %) of nanoreinforcement (carbon nanotubes or nanoclay [56]) in the polymer matrix of composites, fiber sizing or interlaminar layers can allow to increase the fatigue resistance, shear or compressive strength as well as fracture toughness of the composites by 30–80% [57,58]. Loos, Manas-Zloczower and colleagues developed various wind turbine blades with secondary carbon nanoparticles reinforcement (vinyl ester, thermoplasts, epoxy composites containing CNTs) and demonstrated that the incorporation of small amount of carbon nanotubes/CNT can increase the lifetime up to 1500% [59]. Koratkar and colleagues [13,60] studied graphene as a secondary reinforcement for the nanomodification of wind turbine composites, and showed experimentally that the graphene reinforcement is very promising in the development of stronger, long-life turbine blades for the wind industry. Merugula and colleagues [61,62] estimated theoretically that the addition of 1–5 wt % of carbon nanofibers (CNF) to the interfaces of glass fiber reinforced epoxy composites for blades in 2 MW and 5 MW turbines leads to improved tensile stress and modulus, and allows 20% weight reduction of the blades, leading to the increased lifetime. One should note that transferring property improvements obtained in specific polymer-nanocomposites (without fiber reinforcement) as matrix material to laminates with reinforcing fibers remains an issue, especially with respect to volume fraction of the nano-fillers and the lower bound of the scatter in improvements obtained [63]. In some cases, improvements from using nano-modified polymers as matrix (e.g., for improved strength or toughness) come with intrinsically lower property values in other areas (e.g., glass transition temperature) limiting the processability or the applicability of nano-modified polymers [64]. In [65,66,67], the applicability of hierarchical composites for wind energy applications is analyzed, using the computational modelling. Also, the feasibility of using hybrid and nanoreinforced composites in wind blades, as a replacement for the currently used glass fiber/epoxy composites is evaluated in [65]. It was demonstrated in numerical studies that the gains in the lifetime of the composites justify additional investments to produce the wind turbine blades from hybrid and nanoreinforced composites. Still, as noted in [68], there remains a lot of practical and economical challenges before the nanoengineered wind turbines are used. Figure 6 shows a micrograph of a carbon fiber with CNT reinforcements in fiber-matrix interface (a) and computational model of a composite with secondary CNT particles.

### 3.3. Sizing

Fibers for composites are being applied with a surface sizing often referred to as sizing. The application happens during or just after the manufacturing. The two main reasons to apply sizing is to protect the fibers and to increase bonding to the matrix. The adhesion between fiber and matrix is pivotal when it comes to stress transfer between fiber and matrix and the mechanical properties of the composite. The stress transfer happens not only between the fiber and the matrix but across the sizing as well. Unfortunately the sizing and its reactions in the interface is barely understood due to the complexity and inaccessibility opposes thorough investigation. 

Glass fiber sizing is applied as a part of the manufacturing process. After mixing and melting the glass components the melt is lead to a bushing with a large amount of holes from which the fibers are drawn continuously with an impressive speed of 2500 m/min. As soon as the melt leaves the bushing it is rapidly quenched by water being sprayed on the drawn fibers. The quenching ensures the amorphous network structure within the glass that yields the flexible. Several meters below the water spay the fibers pass a roller half immersed in sizing only close enough to pick up the liquid without touching the roller. The sizing contains around 3–10 wt % solid material in an aqueous suspension. After this step the fibers are gathered in a strand often referred to as a roving which contains in the range of 50–4000 single fibers. The fibers are then dried in large ovens above 100 °C for hours in order to evaporate surplus water and for curing of the sizing. Within seconds after the application the effect of the sizing is being put to work in regards to protecting the glass fiber surface against fiber-fiber damage in the rovings and in the later weaving [70,71,72,73,74,75].

The ideal sizing not only protects the otherwise fragile fibers during processing it also reduce fuzzy behavior, it disperse well on the fiber surface resulting in a homogeneous product, it ensure a good wetting during manufacturing of the composite yielding low a low void content, and it maximize the fiber matric interaction for optimum stress transfer. The multiple tasks cannot be covered by one compound thus the need of multiple components [75,76]. The patents behind sizings have been studied and they reveal that sizings for glass fibers consist of minimum a film former and a coupling agent, but mostly more components are included. A large study indicated that the film former makes up around 80 wt % of the dry sizing and the coupling agent around 10 wt %. The task of the film former is to protect against fiber-fiber damage and to protect the roving during winding and weaving. It is often a polymer similar to the matrix that the end-product aims for e.g., polyesters, polyurethanes, and epoxies yielding a good wetting during composite manufacture. Reduction of the stress corrosion triggered by water is attained by the addition of a coupling agent [77].

This is often chosen to be an organosilane and in some cases chromium or titanium oxides. Organosilanes has the possibility to react with the glass fiber surface through a sol-gel reaction which can covalently bond the organosilane or a polymeric form of the organosilane to the fiber surface. With a functionality of the organosilane that complement the matrix it is possible for these to react thus forming a connection between the fibre and the matrix. This is the reason why the coupling agent is considered a crucial parameter in regards to the adhesion between fibre and matrix. The most used silanes have amine or epoxide functionalities. Anti-static agents reduce the fuzziness of the fibres and thereby helps form the roving. Emulsifier agents stabilize the insoluble components that are added to the sizing suspension. Furthermore they reduce the formation of foam and adjust the viscosity of the sizing. Lubricants improve the dispersion on the glass fibres and help protect the surface. Acid or alkalis can be added to adjust the pH to around 4 in order to facilitate the hydrolysis of the silanes. Wetting agents and anti-oxidants can also be added to sizings [73,76,78,79].

Despite that carbon fibers are organic and therefore don’t have the same compatibility issues as glass fibers they still need sizing to manage the smooth and often inert surface. Carbon fibers receive both a surface treatment and a sizing both of which are conducted after the manufacturing of the fibers. The manufacturing of carbon fibers is much different from glass fibers. The steps include polymerization from a precursor, stabilization, and carbonization followed by surface treatment and sizing application. The surface treatment etches or roughens the surface using an anodic or nonionic electrolytic polymer e.g., epoxy, polyamide, polypropylene, or polyurethane dispersions. The result is an increase of the surface area which then increases the bonding between fiber and matrix. An increase in the fiber roughness is also expected to increase the interfacial sliding friction between fiber and matrix after debonding. The amount of sizing is in the range of 0.5–5 wt %. As with the glass fiber sizing it does more than protect the fibers it also facilitate strand formation, reduce fuzz, improve processability and increase bonding between fiber and matrix. The sizing is often an emulsion of polymeric components [80,81,82,83].

Natural fibers are hydrophilic and bond poorly with both thermoplastic and thermoset polymers. Very little work has been done in the field of sizings for natural fibers and using glass fiber sizings is not the best solution. As a coupling agent it is mostly silanes and isocyanate that are selected. Physical treatments as plasma or heating are also used to alter the fiber surface for better bonding to the resin. The treatment is carried out as a part of the processing before being woven. Sometimes the coupling agent is an additive to the matrix resin instead of a surface treatment of the fibers, making it less efficient in composites with low fiber content as modified polymers can hide within resin rich areas with little contact to fibers [84,85]. 

## 4. Testing, Degradation and Computational Modelling

### 4.1. Testing of Wind Turbine Blade Materials and Structures

**Figure 7:**
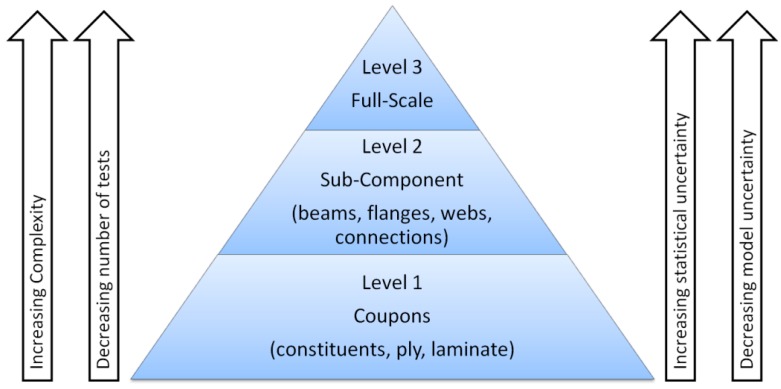
Illustration of type of tests, which can be performed for assessment of load bearing capacity of wind turbine blades.

In the design process of wind turbine blades, tests on several scales can be performed in order to measure the relevant material properties and to check the accuracy of the computational design models used to estimate the load bearing capacity, see Figure 7. However, currently only coupon and full-scale tests are required in the IEC 61400 standard for wind turbines in order to certify wind turbine blades.

At coupon level, small test specimens with the basic material are tested in order to determine the material properties and their statistical characteristics in both ultimate and fatigue limit states. The test specimens at coupon level are normally relatively inexpensive to produce (small amount of materials), are normally relatively easy to model and interpret and with several different tests with many repetitions a good description of variation can be obtained.

At subcomponent level parts of a wind turbine blade are tested in order to determine selected parts load bearing capacity and verify computational models for potential critical details. Subcomponent tests are in general more expensive and complicated to test than coupons for which reason fewer tests are performed with each subcomponent.

**Figure 8:**
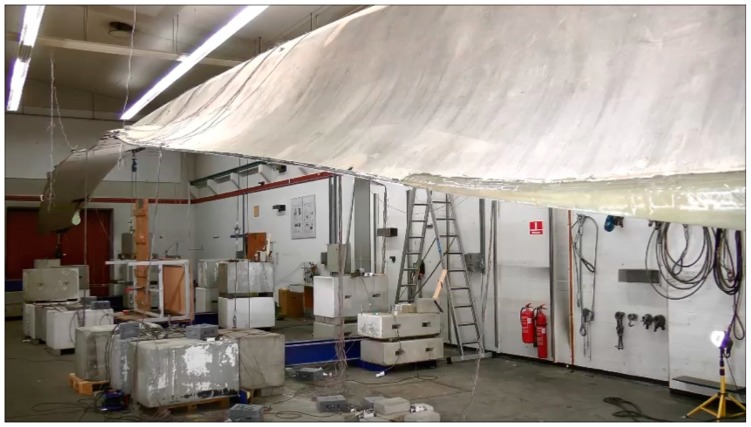
Full-scale testing: A 34 m long wind turbine blade subjected to static test in a combined flapwise and edgewise load direction.

At full-scale level prototypes of the blade are tested both dynamically and statically following the requirements in the IEC 61400-23 standard on full-scale testing [86], see Figure 8. Full-scale blade tests are performed on typically one or two blades in order to verify that the blade type has the load carrying capability and service life provided for in the design. Since the cost of a blade itself is high, the blade is large and usually equipped with a lot of transducers, sensors and instruments, and the time needed for the dynamic test and the subsequent data analysis can be several months for large blades, the cost due to waiting time for market introduction is also significant.

**Figure 9:**
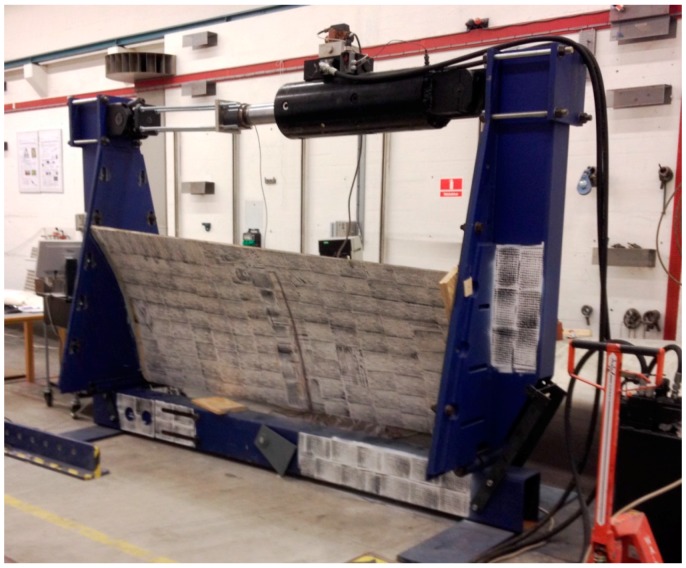
Sub-component testing of trailing edge panel in compression using the method developed at DTU.

Initial work on how to plan and apply subcomponent tests in the design process of wind turbine blades were done in the project: “Experimental Blade Research—Phase 2 (EBR2)” [87,88]. Parts of this work was then used for making the new DNV GL rotor blade standard DNVGL-ST-0376 [89], which for the first time makes it possible to use subcomponent testing as part of blade certification [90]. A subcomponent test method designed to check the compressive strength of the trailing edge region in wind turbine blades under a simplified loading (see Figure 9) were first proposed by DTU [87,91] and then further developed under the EU-funded project IRPWind in corporation with Knowledge Centre WMC [92] and Fraunhofer IWES [93]. In [91] finite element simulations show that the proposed static subcomponent test method is promising in obtaining a test of the compressive strength of the trailing edge region under a simplified loading. It is overall found that the failure load and failure mode is very similar to full blade test for the analyzed test specimen.

Currently the IEC61400-23 and DNVGL-ST-0376 standard require that blade fatigue testing is carried out by testing blades in two directions—flapwise and edgewise—one direction at a time. During their lifetime wind turbine rotor blades are exposed to highly dynamic loads, resulting from cyclic changes in gravity direction, centrifugal forces, and changing wind conditions such as average wind speed, turbulence intensity, rapidly changing wind direction, wind shear, extreme wind gusts and site-specific loads like e.g., wake effects from neighboring wind turbines. The broad and complex load spectrum results in the accumulation of a significant amount of fatigue damage over the turbine lifetime. Fatigue of materials and interfaces are therefore major failure mechanisms in wind turbine blades. The currently required fatigue testing methods are not representing the real service loads very well and there are therefore attempts to develop more realistic test methods. One method is dual-axis fatigue testing where the flapwise and edgewise directions are tested simultaneously [94]. This approach is shown in [95] to be more representative of the loading seen in service and can thus contribute to a potentially more realistic testing of wind turbine blades.

### 4.2. Mechanical Degradation of Wind Blade Composites

The in-plane material degradation of the wind blade composites under loading can start at the microscale cracking, e.g., by fiber failure [96,97]. Once a fiber fails, a local load redistribution occurs. An area with a broken fiber will induce a higher stress concentration in the matrix, which can lead to the matrix cracking. If the fiber/matrix interface bonding is sufficiently weak, cracks form on interfaces (fiber/matrix debonding), leading to the fiber slip along the fiber/matrix interface. This can potentially promote further matrix cracking and debond crack growth. For delamination cracks, if the crack bridging mechanism is operative, the load is shared by the bridging fibers and crack tip, and the stress intensity factor on the crack tip is reduced [98]. A higher amount of bringing fibers leads to the lower stress intensity factor on the crack tip, and the resistance to crack growth increases with increasing the crack length (R-curve behavior). The extension of a crack, bridged by intact fibers, leads to the debonding and pull out of fibers that increase the fracture toughness of the material [99,100]. The fiber debonding, fiber fracture and pull-out are the most important toughening mechanisms in fiber reinforced composites.

In the regions under compressive loading (the downwind side of the blade and spar) fiber crushing and shear banding can be observed. In [101,102], the damage mechanisms of glass fiber composites under compressive loading was studied experimentally, using SEM observations, as well as numerically. The damage mechanisms under cyclic loading (fatigue) are in many cases different from the static damage mechanisms. For multidirectional laminates, the longitudinal plies (with fibers aligned in the direction of tensile load) control the fatigue behavior and lifetime of the composites. The presence of backing fibers (i.e., fibers oriented off-axis to the load direction) can also have a negative effect on the fatigue life of the composites [103]. 

Under tensile cyclic loading along the fibers, damage mechanisms are similar to those in static loading when high tensile strain is applied (so-called “Region I” of the fatigue-life diagram, corresponding to high loads and low lifetimes, see [104]). If small strains are applied, the damage growth is rather slow or even negligible, and does not lead to the material failure even after 108–109 cycles of loading. Under the cyclic loading which is between these two regions, progressive fiber bridged cracking, progressive debond propagation and stochastic fiber breakage are observed in the composites. In laminates with off-axis or backing plies, the damage often begins in the plies oriented transverse to the primary loading direction [105]. In these plies, a crack forms from coalescence of fiber-matrix debonds. When the crack forms in transverse plies, it can lead to the stress concentration in nearby off-axis plies. 

### 4.3. Computational Modeling of Wind Turbine Materials 

The aerodynamically optimal shape of wind blades corresponds to the much lower blade thickness than that dictated by the structural design requirements. However, the structural design of blades is carried out on the basis of safety factors, which should take into account the unknown effects in the loading, degradation, cracking and failure of blades. It is often assumed that the safety factors used in wind blade design are taken too high [5,106]. In order to determine safety factors more precisely, detailed information about the effects of different loading conditions (extreme winds, moisture, temperature variations) and the microstructure parameters of wind blade composites on their strength and lifetime is required. Further important tasks of the analysis of wind blade materials are the prediction of strength and the lifetime of the wind turbine rotor blades, analysis of the effect of different service conditions on the blade performances and analysis of reserves of improvement of the reliability and performances of wind turbines related to the microstructures of the wind blade materials (such as fiber sizing, clustering, nanoscale-engineered matrix, etc.).

This information can be obtained from computational models and theoretical and numerical studies of the wind blade material behavior under loading. For the modelling of damage processes, a series of analytical and numerical methods are used. Among the analytical methods used, one can mention shear lag-based models, fiber bundle model and its generalizations, fracture mechanics based and continuum damage mechanics based models [107]. In the shear lag model, the force balance in a single fiber is considered assuming that the load transfer from fiber to matrix occurs only via shear stresses. In the fiber bundle model, the damage evolution in a bundle of fibers after one or several fibers fail is considered using statistical models of fiber strength and different load sharing rules. Using the shear lag and fiber bundle models, one can simulate the damage evolution in composites. In the continuum damage mechanics, the influence of many microcracks on the material behavior is modeled as tensor characterizing the reduction of the effective areas of a material due to the microcrack growth. In the framework of analytical micromechanics of materials, the relationships between volume content of reinforcements, its shape and orientation and the mechanical properties of materials are established using the variational principles of mechanics of materials and other continuum mechanics methods. These relationships allow studying the effect of the material structure, reinforcement geometry and distribution on the elastic properties of the composites. For the analysis of microstructure-properties relationships of wind blade materials, taking into account complex loading, initial and evolving microstructures (e.g., damage), numerical micromechanical methods are applied. Most often, the problems are solved using the finite element method, in which the solution of integral and differential equations describing the material deformation and microstructure evolution are obtained on the basis of discretization of the considered bodies and discrete approximations of the equations. Multiscale models of wind turbine blades, including also the material degradation at several scale levels, represent an important direction of the wind blade materials analysis [108]. 

## 5. Damage in Operating Wind Turbine Blades: Inspection and Monitoring Tools

Minor damage in the composite can be tolerated if it does not impair the structural performance of the turbine or risk propagating under normal operating conditions. But some forms of damage, once present in the structure, will propagate quickly and reduce the performance of the turbine or even cause an instability that will overload other structural components and potentially cause structural failure of the turbine. Clearly this will halt the operation of the turbine. Once this has occurred the wind farm operator will face a significant cost to make the necessary repairs before the turbine can be put back into operation. Although no strict guidelines exist to determine the criticality of different damages and defects to be found in operating WTBs, all wind farms have an inspection and maintenance procedure for their blades. The justification is that by checking the condition of the structure regularly, it is possible to schedule a series of minor repair tasks that will reduce the financial risk of an unscheduled major repair becoming necessary. 

This process requires firstly an inspection of the current condition of all the blades. Previously requiring direct access by maintenance crew using climbing ropes or a crane lift, this task is more commonly done now with high resolution camera images taken from the ground an inspection platform, or mounted on drones [109]. These images can be handled by computer software that “stiches” all the images together to allow a detailed overview of the entire surface area. Image analysis software can then compare previous inspections to the most recent and highlight any “exceptions” that should be checked by a trained blade engineer. In this way surface cracks (and also occasionally surface dirt and lighting/shading effects) will be registered for more detailed inspection in the follow-up phase.

As mentioned earlier, many severe damages are sub-surfaces and it is not always possible to make a judgement on the severity of the damages via an image. Therefore, a close inspection by a maintenance engineer must also be scheduled. Visiting all the areas highlighted by the initial inspection, the engineer will gather more information on each damage area (perhaps also discovering new ones) and generate a recommendation for each blade’s repair requirement. The inspection is commonly based on the simplest and most robust techniques of visual assessment and manual tap testing. From this effort a job list (and a cost estimate) for the entire wind farm repair will be produced and approved.

It is common for external contractors to bid for the maintenance contract on large wind farms. Although lucrative, it is a competitive business and as the wind industry has grown and matured, access technologies and logistic advances have, together with the competition for contracts, had a lowering effect on the market price. Resisting this is the trend for larger, more complex and damage critical blades being placed in remote offshore wind farms. So maintenance and repair for blades in wind farms is still a significant portion of operating costs. And for operators there is also the consideration of risk around ensuring trustworthy third party inspections and repairs. On-site repairs are routinely documented with an annotated photograph of the pre- and post-repair damage. More analytical evaluation of the repair effectiveness is not conducted.

The idea of introducing a degree of automation into the entire inspection process for wind turbine blades has been investigated for some time [110,111,112,113]. Sensors mounted in or on the blades provide continuous data remotely to the wind farm operator that can then be used to make best use of the available maintenance manpower. But implementing this in operating wind farms would incur additional expense at the highly price-focused manufacturing stage, where embedded sensors would need to be integrated with the structure. And transporting expensive and fragile measurement and inspection equipment to the harsh operating environment of offshore wind farms has similarly limited the uptake of more advanced NDT hardware. Whereas the robust nature of visual/manual inspection (now streamlined and improved by tele- or drone photography and image analysis software) has been favored.

Advanced inspection tools (such as Ultrasonic scanning and thermography) are routinely used in the industry to provide Quality Assurance and Control from blade manufacturing. And full scale blade test facilities use sensor technology (resistance strain gauges, fiber optics, acoustic emission, digital image correlation, etc.) to provide real-time information about the response of the structure to various load conditions, and warnings about the occurrence and severity of any damage events. So there is no question that there exists inspection and monitoring technology that can be applied. The challenge that has yet to be solved is the relevant integration of robust, low-cost monitoring and inspection technology into the entire lifecycle analysis of the large wind farms that provides incontrovertible evidence of an improved exploitation of the structural asset. The discussion on detection tools is a balance between sensors with high sensitivity (enabling very early detection of small damages) possible requiring many sensors with small spatial coverage to sensors with less sensitivity, and thus much later detection of the damage, but requiring less sensors.

The offshore wind sector will continue to expand over the next several years (under 20 GW in 2016 to over 150 GW in 2030); in parallel to this, sensor (and inspection) technology will continue to become cheaper, more robust, with a higher functionality, and more widely applied and integrated within industrial systems and processes. In addition, the materials and structural design and manufacturing (plus repair procedures) will also advance. It seems inevitable that some combination of sensorised multi-functionality will shortly be incorporated into blades that are specifically designed to take advantage of these new understandings emerging around damage detection and damage tolerance criteria [114]. The most commonly investigated sensor technologies for permanent on-line monitoring are described below.

Vibration based damage detection systems rely on analysis of the dynamic response of the blades, either during operation or following an applied mechanical input. Damage identification is commonly based on the comparison between an undamaged and a damaged state. Ideally the ambient energy generated by the turbine operation would be used as the excitation source, however under realistic conditions detecting damage with simple analysis is challenging as the same order of modal property variations are generated by environmental effects and noise contamination [115]. Therefore, more sophisticated methods must be deployed to create a reliable SHM system [116], and this can limit general application. For vibration based damage detection, more success has been reported using an external shaker or embedded actuator as using these a well distributed excitation is created within the entire structure, and a flat spectrum is generated in the frequency range of interest [117]. Vibration based techniques have the advantage of being a mature, well-proven and cheap solution with respect to wind turbine gearbox and bearing fault detection systems. Despite the challenges involved in monitoring the more complex blade structure, some successes have been demonstrated. However, structural response changes will only detect relatively large damages in the blades and this limits the usefulness of the technique.

Fiber optics embedded in the blade structure can be used to measure strain. Several fibre Bragg gratings (FBGs) can be multiplexed within a single measurement fibre optic and with high sensitivity and reliability return local point strain measurements [118]. Combined with their long fatigue life and immunity to electromagnetism, fibre optics are a promising sensor technique for integration within fibre reinforced plastic structures. FBG based systems are the most technologically ready fibre optic measurement system currently with commercial systems reducing prices and the size of installation hardware, as well as improving their robustness. The problem of fragile, bulky and expensive fibre optic measurement hardware is still true for most other (non-FBG) forms of fibre optic systems. For example, distributed sensing based on optical backscatter reflectometry [119,120] recognizes changes in density and composition along the entire length of an unmodified optical fibre. Once recognized by the system, this “fingerprint” value will change due to any local variations in strain and temperature allowing measurements to be returned all along the fibre with a high resolution. This gives far more detail than even a heavily multiplexed FBG array can provide. The technique however, is currently limited to static testing as the extremely low levels of natural back scatter means signal to noise ratio is unacceptable in a dynamic situation.

Acoustic emission (AE) involves detecting transient bursts of elastic stress wave energy released by the formation of damage within the structure. Usually achieved with surface-mounted piezoelectric sensors which transform the elastic energy into an electrical waveform which can be processed and analysed. Detectable AE signals occur when a fibre composite material begins to experience local fiber failure, debonding, matrix cracking, delamination and splitting as the structure is placed under load. FRP structures generate huge numbers of such AE signals when loaded above previously reached maximum loads, as well as at lower load levels when previously formed damage is already present in the structure. This means that AE is a simple, useful and intuitive tool for detecting and locating damage during full scale testing in both static and dynamic loadings. The frequency range of useful AE signals is between 100 kHz and 1 MHz which above that used in vibration-based sensing (and in human hearing), but below the range designated as ultrasonic. Acoustic emission monitoring systems are used commercially in rotating machinery, metallic structures, bridge structures, and simple composite structures like pressure tanks. However, in-operation wind turbine blade monitoring is not a commercial activity due to the expensive hardware, the large data sets generated by the high sampling rate, and the attenuation rate for this frequency range in composite materials limiting the sensoric range meaning many sensors would be required to fully instrument each large wind turbine blade.

Acoustic emission is a passive technique that detects energy generated by the structure, in contrast guided wave technology (or acoustoultrasonics) is the terms used when the piezoelectric sensors are instead used as active transducers to generate a pre-defined input signal that can propagate through the structure and be detected by neighboring sensors [114]. This known “Pitch-Catch” configuration will be altered when the signal interacts with damage or other factors that affect wave propagation between the input and detection points. In this way a network of sensor transducers can monitor any changes occurring over an entire structure. Commercially this technology is successful in simple oil and gas pipeline monitoring, however the complex composite material and dynamic structural environment of the wind turbine blade is proving a challenge. The use of guided wave technology for this application is therefore still in development, but is considered a promising technique.

## 6. Recycling

As pressure is put on to have greener and more sustainable products, the recycling of wind turbine blade have increasingly attracted the interest of wind turbine blade manufacturers and owners. However, recycling blades remains a challenge. The difficulties related to the process are mainly due to the structure of the blade and to the composite materials used. 

The structure of the blade, as represented in Figure 2, is made of several elements, namely shear webs, load carrying beam, leading and trailing edge and aerodynamic shell. Depending on the blade manufacturer, the design and the arrangement of these elements will be different. In general each of these elements is consisting of a specific type of composite and the blade is manufactured as a one-piece component. To separate the different elements, the locations of the elements need to be known and a saw with diamond blade and sufficient water cooling is required. Due to that complex structure, it is difficult to recycle blades into any other application than blade. In addition, the blades to be recycled will be found in various conditions. Decommissioning of wind turbines can be decided as the turbines are reaching end of life, but also at earlier stage if it becomes interesting to replace the turbines by newer models or because the turbines were prematurely damaged. As a result, the quality of the material found in blades and the quality of the blade structure will be varying from blade to blade. The assessment of the blades conditions also represents a challenge. Visual inspection, which is normally used to determine the conditions of blades during inspection, does not reveal the presence of potential sub-surface damages. Finally, the amount of material coming from blades will fluctuate greatly as material will sporadically come from the decommissioning of single turbine or large windfarm. To summarize, the amount of material to be recycled coming from wind turbine blades will be varying in design and material, in quality and quantity. The development of a sustainable recycling solution for blades is therefore very complicated.

**Figure 10:**
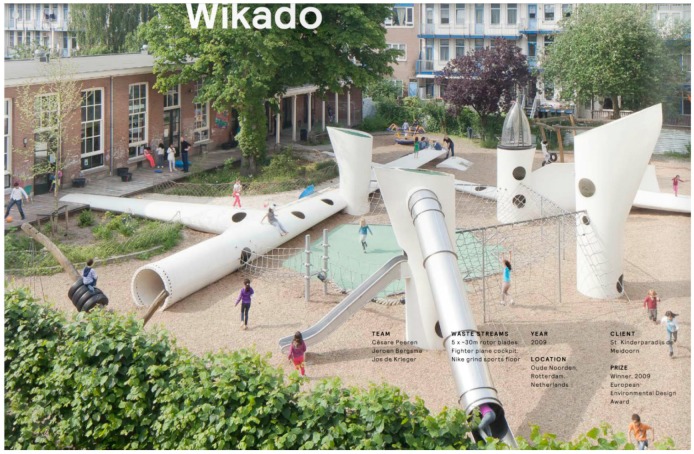
Playground design by the SuperUse studio [129].

The other challenge in recycling blades is related to the composite material used in blades, which are made of a thermosetting matrix and glass fibers or a combination of glass and carbon fibers. Unlike thermoplastics, thermosetting matrix cannot be remolded to form new product. So the options are either to reuse the blade and the composite material elements as they are found in the blade or to transform the composite material into a new source of material. The first option only requires cutting the blade, while in the second option, heavier and more advanced processes need to be use. The first options will necessarily lead to a limited number of possible applications, while the second options will open up to many more. As an example, Figure 10 shows a playground made out of entire sections of blade. The structure of the blade is reused, but the application will be difficult to upscale to an industrial scale solution. Regarding the solutions involving heavier reprocessing, research on composite recycling has focused on processes to separate the fibers from the matrix to reuse them in new polymer composite applications [121]. To do so, heat is necessary to degrade or dissolve the matrix material. The temperatures used in the recycling processes vary from 280 °C for a supercritical fluids process to 450 °C for a fluidized bed process [122,123,124,125,126]. The issue is that the heat treatment will be detrimental to the mechanical properties of glass fibers, which will become extremely brittle [126]. The glass fibers properties are however not the only challenge to overcome. The recovered fibers should also have not too rough surfaces and should be repositioned in specific directions to deserve the purpose of the new application. Finally the cost of the recovered fibers represents the main barrier for implementing these processes on an industrial scale, as pristine fibers remains less expensive. A simpler transformation of the composite material is to shred it. This solution represents however a significant down-cycling of the composite material, as the resulting shredded composite can only be used as a filler or similar material. Neowa GmbH, is using this technique to recycle glass fiber reinforced thermosetting composites in cement production [127]. This company is currently the only industrial recycling station in Europe able to process composites. Another solution based on shredded composite was developed by Miljøskærm in Denmark. The company uses agglomerated shredded composite in sound insulation panels [128]. Given the challenges presented, it seems that a sustainable solution for recycling wind turbine blade will need to be based on a combination of several solutions in order to consider all possible scenarios.

## 7. Conclusions

For the reduction of the fossil fuel dependency, the renewable energy, in particularly, wind energy production should be drastically expanded in the next decades. This can be achieved by the installation and use of large and extra-large wind turbines, to be placed in wind parks either off-shore or on-shore. The basic requirements to the performances of such wind turbine can be satisfied only by using advanced, lightweight, highly durable, fatigue resistant and damage tolerant and stiff composite materials. 

The most important parts of the turbines, produced from composites, wind turbine blades, are subject to complex, combined impact, static and random cyclic loading. In order to resist these loading over many years and hundreds of millions of loading cycles (on the one side) and to reduce the loads (like gravity, on the other side), the wind blades are built from fiber reinforced polymer composites. While the currently available solutions (in the simplest case, E-glass/epoxy composite) satisfy most of these conditions partially, the necessity for new, better solutions leading to the increased reliability and reduced costs for wind turbines, is apparent. That is why a lot of efforts are put in the development of new, stronger, more damage resistant, faster producible, more environmentally friendly and recyclable composites for wind turbines. Some of the promising directions of development of stronger, more reliable, environmentally friendly and economically producible composites are listed below. 

Development of new epoxy resin systems which have low mix viscosity, better wetting of fibers (by modifying either resins or applying special sizing on fibers) and allow low infusion pressure in the vacuum assisted resin transfer molding (VARTM) should lead to the blades with minimum production defects. Further, automated component deposition during VARTM can allow improving the wind blade quality as well. Yet, the increase in size of turbine blades most likely leads to more manufacturing defects. Thus, the development of more damage tolerant materials is desired. Resins with faster cure and lower curing temperature allow reducing the processing time and automating the manufacturing.

Carbon fibers represent a very promising alternative to the traditional E-glass fibers. Other alternatives are high strength glasses, basalt, aramid and natural fibers. Carbon fibers ensure higher stiffness while their disadvantages are higher costs, lower compressive strength and high sensitivity to local defects (e.g., misalignment). In several studies, the combination of carbon and E-glass fibers was recommended as a promising solution, which allows to achieve the combination of higher stiffness (due to carbon fibers) with limited cost increase. With view of resin matrix, thermoplastics have some advantages over traditionally used thermosets, e.g., recyclability. The investigations on the applicability of these groups of materials for wind blade composites have been carried out intensively during the last years. 

The strength and durability of wind blades are controlled by damage processes at the microlevel, in fibers, on the fiber/matrix interfaces, between plies. It suggests an idea that if these microscale properties of the materials are enhanced, the strength and lifetime of the composites, and, generally, wind turbines is increased. This can be realized by nanoscale modifications of the material structures, i.e., by introducing nanoscale particles (of the size order 1–10 nm) in the fiber sizing, matrix and interfaces between plies. The materials with nanoengineered matrix (or sizing) and microscale (e.g., carbon fiber) reinforcement can demonstrate in some cases the up to 80% higher fracture toughness and lifetime than the neat composites.
